# Application of Acoustic Telemetry to Assess Residency and Movements of Rockfish and Lingcod at Created and Natural Habitats in Prince William Sound

**DOI:** 10.1371/journal.pone.0012130

**Published:** 2010-08-13

**Authors:** Brad F. Reynolds, Sean P. Powers, Mary Anne Bishop

**Affiliations:** 1 Department of Marine Sciences, University of South Alabama and Dauphin Island Sea Lab, Dauphin Island, Alabama, United States of America; 2 Prince William Sound Science Center, Cordova, Alaska, United States of America; University of Zurich, Switzerland

## Abstract

Loss and/or degradation of nearshore habitats have led to increased efforts to restore or enhance many of these habitats, particularly those that are deemed essential for marine fishes. Copper rockfish (*Sebastes caurinus*) and lingcod (*Ophiodon enlongatus*) are dominant members of the typical reef fish community that inhabit rocky and high-relief substrates along the Pacific Northwest. We used acoustic telemetry to document their residency and movements in the nearshore waters of Prince William Sound, Alaska in order to assess use of created reef habitat in an individual-based manner. A total of 57 fish were surgically implanted with acoustic transmitters. Forty-five fish were captured and monitored in three habitats: artificial reef, low-relief natural reef, and patchy high-relief natural reef. Within each habitat, both rockfish and lingcod exhibited long periods of residency with limited movements. Twelve rockfish were captured at the natural reefs and displaced a distance of 4.0 km to the artificial reef. Five of the 12 rockfish returned within 10 d of their release to their initial capture site. Another five of the 12 displaced fish established residency at the artificial reef through the duration of our study. Our results suggest the potential for artificial reefs to provide rockfish habitat in the event of disturbances to natural habitat.

## Introduction

Among the major challenges that coastal scientists face are the quantification of impacts of natural and anthropogenic stressors on coastal communities (both societal and ecological), determination of the causes and responsible mitigation of such impacts, and the development of sound restoration strategies to rehabilitate ecological communities. Legal and societal mandates to compensate for degradation of natural resources on land and in the sea have led to dramatic growth in the practice of restoration such that restoration ecology is now a dominant focus of environmental science [1], [2]. Traditionally, quantification of ecological damage and potential benefits of restoration have been based on the presence/absence of key indicator species or by changes in community structure as measured by point measurements of the relative abundance of animals or plants [e.g., [3], –[5]. Although such approaches allow changes in several community metrics to be assessed, they ignore changes in animal behavior that may be equally important in structuring communities [6].

Rocky nearshore areas are essential fish habitat for two demersal fish species, copper rockfish (*Sebastes caurinus*) and lingcod (*Ophiodon elongatus*). Both copper rockfish and lingcod occur only along the coastal waters of the Pacific Coast of North America [7]. In Alaska, both species are highly sought after by recreational fishers, and in the case of lingcod by commercial fishers as well. Both copper rockfish and lingcod are prone to overfishing because they are relatively sedentary [8]–[13] and inhabit nearshore, high-relief habitats that are easily identifiable on nautical charts to fishers. At the same time, both species are relatively long-lived and in the case of rockfish, late-maturing [14]. Maximum age for lingcod is about 25 years and for copper rockfish is 50 years [15], with a typical age at sport harvest for copper rockfish between 10–30 years [16]. Reproduction of both rockfish (as a group) and lingcod is characterized by a pattern of infrequent and irregular years with successful recruitment [17], [18].

The associations of rockfish and lingcod with the nearshore zone also make these fish vulnerable to pollution events including oil spills. Following the 1989 Exxon Valdez oil spill, oil lingered for several years in the sediments of oiled nearshore areas. Rockfish were identified as a species group injured by the 1989 oil spill [19]. Thus, the combined factors of susceptibility to overfishing, low annual productivity, and their association with the nearshore zone make for a low resilience for local area populations of copper rockfish and lingcod in the event of a major disturbance.

The ability of fisheries managers to restore damaged and stressed fish habitat may be vital to a sustainable fishery. One potential tool for restoring habitat following a disturbance is the deployment of artificial reefs to increase habitat complexity and carrying capacity for demersal fish in areas at, or adjacent to, damaged habitat. Artificial reefs are commonly deployed in sub-tropical and tropical marine waters for the purpose of enhancing fish abundance, or restoring habitat following the degradation or loss of natural structure to anthropogenic or acute natural events [4], [20], [21]. However, limited data are available on the function of artificial reefs in sub-Arctic marine waters. In spring 2006, the National Marine Fisheries Service of the National Oceanic and Atmospheric Administration (NOAA) installed Alaska's first preplanned artificial reef near Whittier in northwestern Prince William Sound. The artificial reef was part of a mitigation settlement and was installed to evaluate its potential as a tool for fish habitat restoration and enhancement in sub-Arctic marine waters. Along with Cordova and Valdez, Whittier is one of three major ports in Prince William Sound. Whittier serves as the gateway to the Prince William Sound for the Anchorage metropolitan area and is a port destination for cargo vessels, cruise ships, commercial fishing boats, and recreational vessels. As commercial and recreational development pressures continue in and around the Whittier area, the coastal and nearshore habitats adjacent to Whittier are increasingly stressed and vulnerable to the effects of development and pollution.

Habitat restoration requires detailed knowledge of targeted fish species residence times, movements, and habitat utilization. The goal of this study was to use acoustic telemetry to assess residency, movements, and site fidelity by copper rockfish and lingcod at the artificial reef and at natural habitats in the nearshore waters of Prince William Sound. Recent advances in miniaturization of acoustic tags as well as continued reduction in the cost of autonomous hydrophones have allowed detailed investigation of behavioral patterns of marine fishes [e.g., [22]]. The results of this study are intended to inform fisheries managers on the efficacy of artificial reefs to provide fish habitat in Alaska's nearshore waters and, in a broader context, examine the use of acoustic telemetry in assessing the behavioral response of animals to restored habitats.

## Results

### Tagging and Recaptures

We captured and tagged 27 fish between 26 June and 13 July 2007. From 17 to 23 August 2007 we captured and tagged an additional 31 fish. In all, we tagged 40 copper rockfish, 2 dusky rockfish (*S. ciliatus*), 6 quillback rockfish (*S. maliger*), 4 yelloweye rockfish (*S. ruberrimus*), and 6 lingcod across the three areas (Table 1). One tag inserted into a dusky rockfish failed immediately after insertion and was therefore not monitored.

**Table 1:** Number and total lengths (mm; 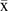
±se) of rockfish and lingcod acoustic-tagged at the artificial and natural reef sites in Passage Canal, summer 2007.

		Artificial Reef			Rock Slide			Bush Banks	
		Total length (mm)			Total length (mm)			Total length (mm)	
Species	*n*	± se	range	*n*	± se	range	*n*	± se	range
Copper Rockfish	3	309±21	285–351	5	306±16	271–345	32	321±6	258–393
Quillback Rockfish	1	280		3	294±20	262–330	2	288±33	255–320
Yelloweye Rockfish	0			1	305		3	488±40	432–565
Dusky Rockfish	0			2	355±15	340–370	0		
Lingcod	0			2	510±46	464–555	4	512±21	460–550

Three copper rockfish and one yelloweye rockfish were recaptured in summer 2007 during hook and line surveys at Bush Banks pinnacles 1 and 2. Days since initial tagging ranged from 36 to 51 d. Visual inspection of the area of incision on each fish determined complete closure for each incision with no evidence of infection. In addition, underwater dive surveys conducted monthly (June through September 2007) as part of the NOAA artificial reef colonization survey observed several tagged rockfish (identifiable by external floy tags) at each of the study sites.

In August and September 2008, two tagged copper rockfish were recaptured by sportfishers at Bush Banks. Both rockfish were initially tagged on 18 August 2007 at Bush Banks, and released at the artificial reef for the homing experiment. The total lengths of each fish at initial capture were 315 and 330 mm and reported lengths at recapture one year later were 350 and 356 mm.

### Residency and Movements

**Figure 1:**
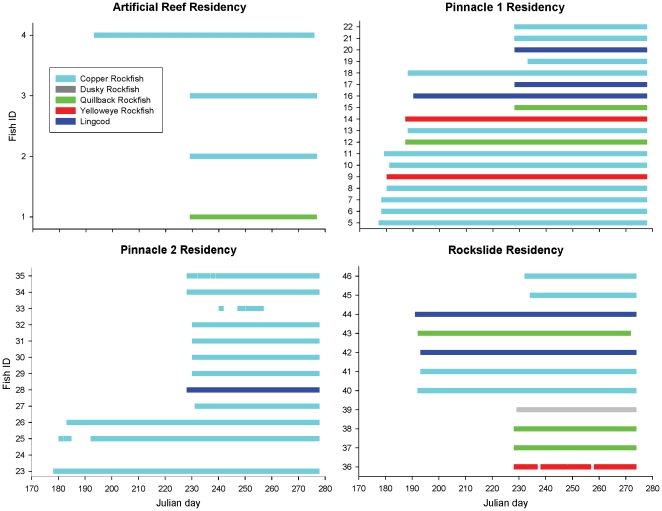
Length of residency at artificial and natural reef sites for acoustic-tagged fish. Fish were captured 26 June to 13 July and 17 to 23 August 2007 and monitored through 4 October 2007. Julian day 177 = 26 June; Julian day 277 = October 4.

We obtained residency and movement data for 45 tagged fish. The majority of tagged fish (96%) demonstrated residency at their tagging sites for the duration of the study, approximately 14 weeks for individuals tagged in early summer, and seven weeks for fish tagged in mid-summer (Fig. 1). At the rock slide, six of the 11 tagged fish were detected variably by each of two receivers indicating a small range of lateral movement along the shoreline. One yelloweye rockfish tagged at the rock slide (#36) moved beyond the array on three occasions with absences ranging from 40 to 73 h. In addition, one copper rockfish (#25) moved beyond the Bush Banks pinnacle 2 array for a 28 h period shortly after initial release, and again for 9 d before returning to the array for the duration of the study. Three fish were detected moving between Bush Banks pinnacles 1 and 2. One of these fish, a lingcod (#28) moved only once to pinnacle 1 for a 14 hour period. One copper rockfish (#35) moved to pinnacle 1 on six occasions for periods of 10 h to 2 d. Another copper rockfish (#33) moved between pinnacles on five occasions, residing equally between each for periods of 1 h to 7 d before moving beyond the arrays after 24 d in the Bush Banks study area. Shoreline transects with the portable hydrophone detected none of the 45 fish outside of the three study areas during 24 August and 27 September 2007 surveys.

### Homing Experiment

**Figure 2:**
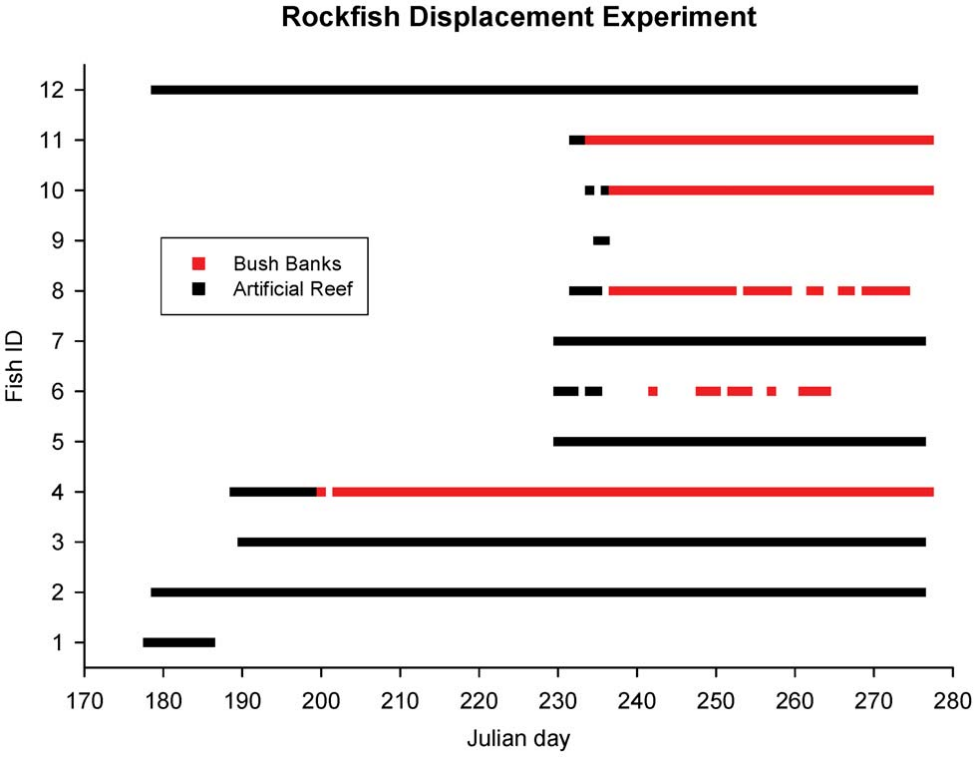
Detections of rockfish following displacement from capture sites to the artificial reef. All fish were copper rockfish excepting one yelloweye rockfish (#9). Julian day 177 = 26 June; Julian day 277 = October 4.

We captured 10 copper rockfish and one yelloweye rockfish from Bush Banks and one copper rockfish from the rock slide site and immediately after tagging, released them at the artificial reef (Fig. 2). Five copper rockfish from Bush Banks resided at the artificial reef for 1 to 10 d (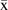 = 3.9 d) before returning the 4.0 km to Bush Banks. Return times (from last detection at the artificial reef to first detection at Bush Banks) ranged from 23 h to 7 d (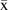 = 2.3 d). One of the five, fish #6, returned to Bush Banks after 5 d at the artificial reef and was detected intermittently for 23 d before disappearing. Two displaced fish, a copper rockfish and yelloweye rockfish resided at the artificial reef for 9 d and 2 d before departing. Subsequently, neither fish was detected by receivers at any of the three study sites. The five remaining copper rockfish, including one initially captured at the rock slide, established residency at the artificial reef for the duration of the study. Length of stay for these five fish ranged from 97 d (June cohorts, *n* = 3) to 46 d (August cohorts, *n* = 2). On 24 August, shoreline transects using the portable receiver detected three fish between the artificial reef and Bush Banks. All three had been displaced from Bush Banks to the artificial reef between 18 and 23 August, and all three returned to their capture site. A paired t-test comparing mean total length of copper rockfish that established residency at the artificial reef with the mean total length of copper rockfish that returned to Bush Banks found no significant difference (*P*>0.05).

## Discussion

The residency and movements we observed for rockfish and lingcod in nearshore areas of our Prince William Sound study site were similar to results published for these species in Washington, British Columbia, and southeast Alaska [8], [9], [11], [12]. The majority of tagged fish (96%) did not move beyond detection of study site arrays, and site fidelity was similar among sites. For 80% of tagged fish, transmitter detections within an array were simultaneous among receivers indicating that fish resided in the area of receiver overlap. This behavior suggests that movements of fish in our study area were central to each site array, and likely confined within small areas of high quality habitat such as pinnacle tops, artificial reef structures, and high-relief substrate. Matthews [9] utilized active tracking and directional hydrophones to estimate home range sizes of copper and quillback rockfish. She documented small (<10 m^2^) home ranges at high-relief reefs and larger home ranges (<0.004 km^2^) at low-relief and patchy, high-relief reefs. The receivers used in our study were autonomous, omni-directional hydrophones and recorded only tag presence. Therefore, fine scale resolution of fish movements were not assessed, and home ranges sizes could not be determined. During this study, fish that maintained residency at the artificial reef exhibited the least range of movements. Detections for all fish occurred simultaneously at the three receivers in the array, indicating that movements were confined to the area of receiver overlap. At Bush Banks pinnacle 1, all 18 fish maintained residency during the study. The largest movements we detected were by fish captured at Bush Banks pinnacle 2 that periodically moved 0.5 km across deeper (35 to 55 m) waters to pinnacle 1.

These results suggest that although site fidelity was similar among study sites, movements within sites were related to habitat complexity. Fish at our uniformly, high-relief site (artificial reef) showed the least movement compared with both the patchy high-relief (rock slide) and the low-relief (Bush Banks) habitats. We suggest that the artificial reef, compared with the other two study sites, constituted a relatively small (3,000 m^2^), high-relief habitat patch. With no similar habitat in proximity, rockfish were less likely to move. In comparison, the rock slide covered a larger geographical area (8,000 m^2^), with patches of high-relief habitat 10 to 20 m apart along the shoreline. Bush Banks pinnacles were both uniformly low-relief habitats, each covering approximately 7,800 m^2^. The movements by three fish from pinnacle 2 to pinnacle 1 suggest that habitat at pinnacle 1 was higher quality. Furthermore, this behavior implies that although copper rockfish and lingcod typically display site fidelity, movements beyond home sites may be made to optimize foraging.

The majority of the fish we tagged were probably subadults. The total length of lingcod captured in our study ranged from 460 to 555 mm with a mean length of 511 mm. Conical papillae adjacent to the anal vent, a physical characteristic of mature male lingcod, were not apparent. In Alaska, the published minimum size (TL) at maturity is 680 mm for females [23], while size at maturity for males is not well-established. For copper rockfish, the average total length in our study was 319 mm (max = 393 mm). Length at maturity for 50% of male and female copper rockfish ranges between 330 to 356 mm [24] indicating that the majority (74%) of fish we tagged were probably not sexually mature.

Although our tagging data indicate high site fidelity during the study period, field surveys suggest a seasonal movement into the area. Hook and line surveys and NOAA SCUBA dive transects found low rockfish and lingcod densities between 26 June and 13 July [25]. By late July, higher densities during these same surveys suggested that increased numbers of lingcod and rockfish moved onto shallow water reefs. Similarly, in British Columbia, two-year-old lingcod recruit into reef habitats during summer months [26]. For copper rockfish, seasonal movements have been documented for some habitats. Matthews [27] observed consistent densities of adult rockfish at high-relief rocky reefs throughout the year. However, adult rockfish summering at low-relief reefs moved to high-relief artificial reefs in fall. She attributed these movements to the loss of habitat complexity at low-relief reefs following algal senescence.

Our homing experiment provided insight into both homing ability and habitat quality. Although rockfish frequently inhabit small home ranges, Matthews [27] suggests that homing in rockfish allows for exploration of adjacent habitats and the ability to return to homesites if more suitable habitat is not found, thus optimizing habitat selection. In Puget Sound, six of seven displaced rockfish returned to their original high-relief habitats following release at low-relief reefs [9]. In our study, five of 12 displaced rockfish returned to their original, low-relief pinnacle following release at the high-relief, artificial reef. This could be interpreted as high fidelity behavior for those individuals. Another five copper rockfish, however, exhibited no post-release movement from the artificial reef back towards their capture site. Instead, these copper rockfish established residency at the artificial reef for the duration of the study. This selection of an alternative habitat may be interpreted as an indication of the potential for the artificial reef to provide quality habitat.

We did find evidence of between-year site fidelity. In late summer 2008, sportfishers recaptured two copper rockfish (#6 and #7) at Bush Banks. Interestingly, while both were originally captured at Bush Banks, both had been released at the artificial reef for the homing experiment. During the experiment, one fish returned to Bush Banks while the other remained at the artificial reef through September 2007 when our monitoring ended. The degree of intra-annual site fidelity by rockfish, and the potential for inter-annual site fidelity has implications for fisheries management. Furthermore, their association with habitat features easily identifiable to fishers (i.e., pinnacles, artificial reefs) increases their vulnerability to fishing pressure. Fidelity to high-relief structure necessitates caution in how management utilizes artificial reef deployments. Artificial reef complexes are generally small in size when compared to natural reefs, and could, therefore, concentrate large densities of fish in a relatively small area.

High densities of fish may be attracted to artificial reefs because they mimic high-relief habitats and provide physical refuge. In the case of rockfish and lingcod, habitat associations for these species indicate a behavioral preference for high-relief structure throughout much of their life cycle [11], [27], [28]. For example, following deployment of the artificial reef at our study site, lingcod and rockfish were immediately found inhabiting the reef [25]. Nevertheless, it may take several years to decades before artificial reefs provide high quality foraging habitat. Initially, artificial reefs lack macrophyte and invertebrate communities. And in the case of sub-Arctic waters, long-term colonization events have not yet been described.

Following pollution events or habitat degradation (e.g., shoreline development, dredging) nearshore habitats may be damaged to an extent that inhibits their ability to support local fish populations. Artificial reefs may be a suitable restoration tool for enhancing damaged habitats. Placement of artificial reefs at, or adjacent to, the site of disturbance may immediately provide refuge, and in time, a suitable forage base. Rockfish exhibit the ability to seek quality forage habitat during peak production season prior to returning to habitats providing physical refuge over winter [27]. Starr [13] noted high site fidelity for adult lingcod, but observed frequent absences suggesting that foraging often occurred away from the site. These findings indicate that replacement of damaged refuge habitat with artificial reefs may be effective.

In conclusion, the establishment of residency at the artificial reef by nearly half of the displaced rockfish and the high site fidelity exhibited by the four rockfish captured, tagged, and released at the artificial reef suggest that the artificial reef was effective in providing rockfish habitat. Additionally, fish utilization data from the concurrent NOAA study of the artificial reef suggest that the reefs are well utilized by both lingcod and several species of rockfish. These results demonstrate the potential for artificial reefs to provide alternative habitat if natural subtidal habitat is damaged. At the same time, we propose that the quality of artificial reefs as productive foraging habitat in sub-arctic waters merits further investigation.

## Methods

### Ethics Statement

Capture, handling, and tagging procedures were approved by the University of South Alabama's Institutional Animal Care and Use Committee (IACUC Protocol 05045 issued to Sean P. Powers).

### Study Area

**Figure 3:**
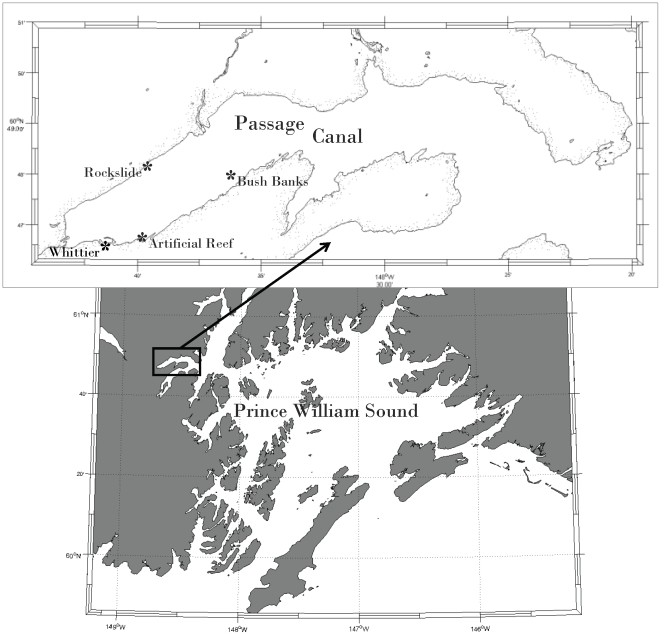
Lingcod and rockfish tagging and monitoring areas at Passage Canal, Prince William Sound, Alaska.

Our study took place in Passage Canal, located in the northwest corner of Prince William Sound, near the port city of Whittier (Fig. 3). Within Passage Canal, we used three study areas that were already established as part of an ongoing NOAA study to evaluate artificial reefs as an enhancement tool for nearshore fish habitat. The three areas in Passage Canal include 1) Smitty's Cove, adjacent to the Whittier Boat Harbor and the location of the artificial reef, 2) Bush Banks, and 3) rock slide. Bush Banks and the rock slide are both natural reef habitats. Seafloor depth adjacent to the study areas typically exceeded 180 m within 0.5 km of each study site.

The study site at Smitty's Cove is relatively shallow (15–20 m deep) and is characterized by a gently declining slope and a mixed, soft and hard sediment substrate. The ∼3,000 m^2^ artificial reef complex includes two, parallel patches of 1 m-high concrete pyramidal Fish Havens© and 1 m-high spherical concrete Reef Balls© deployed in three, paired-plots of 30 reefs each. At the time of the May 2006 deployment, a macrophyte community dominated by *Agarum clathratum* (sieve kelp) and *Laminaria saccharina* (sugar kelp) covered approximately 20 percent of substrate adjacent to the artificial reef. When this study began in June 2007, the macrophyte community was enhanced by *L. saccharina* colonization of artificial reef surfaces. Reef Ball and Fish Haven surfaces were approximately 70 and 15 percent colonized, respectively [25].

Our study site at Bush Banks includes two pinnacles. Pinnacle 1 rises from the seafloor to a depth of 15 to 22 m, and pinnacle 2 rises to a depth of 15 to 25 m. Each pinnacle has an areal surface of 7,000 to 8,000 m^2^, and is characterized by a rocky substrate, also dominated by *L. saccharina* and *A. clathratum*. The distance between pinnacles is 0.5 km, and the distance between Bush Banks and the artificial reef at Smitty's Cove is 4.0 km. Our third site, the rock slide, is a subtidal boulder field adjacent to the shoreline on a 45° declining slope. The total area of high-relief substrate at the rockslide is ∼8,000 m^2^. Vegetation cover is 70 percent and is dominated by *L. saccharina* and *A. clathratum*. The rock slide is 2.5 km from Bush Banks and 3.2 km from the artificial reef at Smitty's Cove.

### Fish Tagging

We used hook and line to capture rockfish and lingcod at our three study sites. We minimized the potential for barotrauma by fishing at depths <20 m, using only barbless hooks, and reeling captured fish slowly to the surface. Fish displaying signs of barotrauma (e.g., everted stomachs, protruded eyes), parasitism, or other signs of ill health were not tagged. Upon capture, we placed each fish in a 10 gallon plastic aquarium containing a solution of ambient seawater and 3-aminobenzoic acid-ethyl-ester-methane sulfate (Ms222; 5 g:1 L H_2_O), an anesthetic. We removed each fish from the solution when it became motionless, placed it on a clean, disposable plastic surgical mat and pumped seawater through the fish's mouth and out through the opercular cavity.

For tag insertion, we made a small incision (1.5 cm) in the abdominal cavity. A Vemco series V9-2H acoustic transmitter (Vemco, Halifax, Nova Scotia) with an estimated tag life of 95 d measuring 9 mm (diameter) ×30 mm and weighing 5 g was placed below the stomach, against the abdominal cavity. The incision was closed with two sutures and swabbed with a broad spectrum antibiotic ointment. Surgery took less than 3 min. We also measured total length (mm) and tagged each fish with an external t-bar tag (46×2 mm) anchored below the dorsal ray. Following surgery, fish were held for recovery in a 20 gallon plastic aquarium with ambient seawater until equilibrium (upright swimming) and active swimming were observed. Recovery was typically observed within 2 to 10 min. Post recovery we released fish in the central part of the acoustic hydrophone array at the capture site.

In order to determine rockfish homing ability and further assess site fidelity, a homing experiment was conducted within the broader residency study. For this experiment, 12 rockfish captured from natural reef study sites were implanted with acoustic transmitters then released into the central part of the artificial reef hydrophone array, 3.2 to 4.0 km from their home sites. Each fish was monitored to determine period of residency at the artificial reef, time of departure, and elapsed time between their departure and return to their original natural reef capture site.

### Fish Tracking System

**Figure 4:**
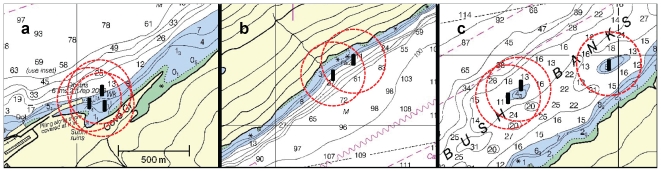
Acoustic receiver array designs and theoretical detection radii. a) artificial reef, b) rockslide, and c) Bush Banks pinnacles 1 and 2. Soundings in fathoms.

We deployed eight, Vemco VR2W hydroacoustic receivers at the three study sites from 26 to 28 June 2007 (Fig. 4). At the rock slide, two receivers were installed 150 m apart, and at opposite ends of the subtidal rock slide. At Bush Banks pinnacle 1, two receivers were installed 70 m apart and at opposite ends of the pinnacle's periphery. One receiver was installed at the western edge of Bush Banks pinnacle 2, a distance of 0.5 km from pinnacle 1. At the artificial reef, three receivers were deployed 65 m apart in a triangular array. One receiver each was centered in the Reef Ball and Fish Haven artificial reef complexes and a third receiver was positioned near the shore of Smitty's Cove. All receivers were moored approximately 1 to 2 m above the seafloor and attached to a 30 kg concrete mooring that had a small, subsurface float. Receiver placements at each site were dictated by bathymetry and location of dive transects surveyed during NOAA artificial reef surveys. With the exception of Bush Banks pinnacle 2 where we deployed only one receiver, the area of receiver overlap at each site was central to each reef.

Tags were programmed to transmit an individually-encoded signal at random, 30 to 90 s intervals. We determined tag detection range by attaching an acoustic transmitter to a weighted fishing line and lowering the tag to a depth of 12 m below the research vessel. At time ‘zero’, the vessel was positioned directly over a receiver moored at 12 m depth. The distance between the research vessel and the receiver was then increased by 10 m increments at 3 min intervals. Based on repeated range tests, the effective transmitter detection distance was estimated at 200 m. Maximum detection areas for each array were estimated at 0.17 km^2^ (artificial reef), 0.19 km^2^ (rock slide), 0.15 km^2^ (pinnacle 1), and 0.13 km^2^ (pinnacle 2). In order to detect movements of tagged fish beyond the hydrophone arrays, a portable Vemco VR100 receiver with an omni-directional hydrophone was towed from the research vessel during shoreline transects on 24 August and 27 September 2007. Each transect followed the northern and southern shorelines of Passage Canal from the head of the canal to the mouth and maintained a 175 m distance from the shore.

Receivers were retrieved and data uploaded using Vemco VUE ® software on 12 July 2007 and at the conclusion of the study between 30 September and 4 October 2007. Transmitter data were analyzed individually, and false transmitter detections in the data set resulting from transmitter collisions or acoustic interference were rejected using criteria established by Vemco [29]. Total residence time was measured by the persistence of a transmitter signal at a study site over time. A fish was assumed to be a resident if it was recorded more than twice in a day at the study site until the termination of our study.

Movements within a study site were determined by variability in transmitter detection between receivers within each study site array. For example, fish simultaneously detected by each receiver in an array were categorized as residing in the area of receiver overlap. If a fish was detected solely at one receiver, then solely at another in the array, such a pattern of detection would indicate that the fish moved across the area of receiver overlap. At Bush Banks pinnacle 2, the placement of one receiver at this site precluded us from detecting movements within the site.
